# Use of Team-Based Learning Pedagogy to Prepare for a Pharmacy School Accreditation Self-Study

**DOI:** 10.3390/pharmacy9030148

**Published:** 2021-08-27

**Authors:** Ruth Vinall, Ashim Malhotra, Jose Puglisi

**Affiliations:** 1 Department of Pharmaceutical & Biomedical Sciences, College of Pharmacy, California Northstate University, Elk Grove, CA 95757, USA; ashim.malhotra@cnsu.edu; 2 Department of Basic Sciences, College of Medicine, California Northstate University, Elk Grove, CA 95757, USA; jose.puglisi@cnsu.edu

**Keywords:** accreditation, team-based learning, team activities

## Abstract

Ensuring adequate engagement and preparation of all stakeholders in an accreditation self-study can be challenging for many reasons, including lack of motivation and inadequate understanding of expectations and procedures. The goal of this exploratory study was to determine whether using team-based learning (TBL) pedagogy to deliver an accreditation preparation workshop could effectively prepare and engage participants. A Likert-scale questionnaire was administered to workshop attendees (*n* = 52) to determine whether they found TBL-based training helpful and whether it promoted engagement. Twenty-four attendees completed the survey (46%). More than 80% of participants strongly agreed or agreed with 12 statements relating to perceptions of self and participant engagement within team activities and the usefulness of team activities. More than 65% of participants strongly agreed or agreed with statements relating to the helpfulness of the TBL approach in preparing for the self-study (five questions). Subgroup analysis showed no significant difference in responses based on whether on not participants had previously been involved in an accreditation self study. Our data indicate that a TBL approach can be an effective way to engage and prepare stakeholders for an accreditation self-study, and that TBL pedagogy has utility outside of the classroom setting.

## 1. Introduction

Health profession education (HPE) programs such as those leading to professional doctoral degrees in pharmacy, medicine, psychology, occupational therapy, nursing, and others must deliver standardized and high-quality education to their graduates. Such education, at a minimum, must satisfy a plethora of highly specialized and specific theoretical, practicum-based, and clinical quality standards, typically enshrined in HPE program accreditation [1,2]. Since HPE graduates immediately impact the communities they serve, most HPE programs must strictly adhere to and demonstrate achievement of all mandated accreditation standards [3,4,5]. In the United States, compliance with accreditation standards is typically documented through completion of a self-study in which HPE programs must work with all stakeholders including administrators, faculty, staff, students, preceptors, and alumni to generate a self-study report, which is then reviewed by accreditation agencies prior to a site visit [6,7,8]. A successful accreditation self-study report and site visit is quintessential to the vitality and sustainability of pharmacy programs not only in terms of an independent assessment of program deliverables, outcomes, and the overall quality of the graduate, but also in terms of program reputation and viability.

It follows that HPE programs must optimally effectuate the accreditation self-study in an efficient, timely, and inclusive manner. Doing so requires stakeholders to have a good understanding of the accreditation self-study process and a good working relationship with fellow stakeholders. In the context of pharmacy education, Doctor of Pharmacy programs in the United States are accredited by the Accreditation Council for Pharmacy Education (ACPE). Doctor of Pharmacy programs are required to show compliance with 25 standards that comprise three main areas: educational outcomes, structure and process to promote achievement of educational outcomes, and assessment of standards and key elements. Extensive guidance is provided by ACPE regarding what evidence and information must be provided in the self-study report [3]. Evidence of engagement of all stakeholders in the self-study process is also required and must be documented in the first page of the accreditation self-study report.

Ensuring adequate understanding of the ACPE accreditation requirements and engagement of all stakeholders can be a major challenge due to the complexity of the process and the varied background and experience of stakeholders. In addition to addressing ACPE requirements, additional layers of complexity are added due to the need to align the self-study report with local, state, and regional accreditation guidelines as well as ACPE guidelines [9,10]. There are several peer-reviewed publications that outline strategies which Doctor of Pharmacy programs have employed to help promote the success of an ACPE accreditation self-study. For example, Philips et al. described their approach for promoting widespread stakeholder engagement in the ACPE accreditation self-study process. Their suggestions for success included creating a self-study communication plan to promote stakeholder engagement and feedback, and use of a timeline of involvement to ensure regular feedback is solicited [11]. Evans et al. and Timpe et al. have both advocated for the implementation of continuous quality improvement to help ensure a successful pharmacy accreditation self-study and to make the self-study process more meaningful and help increase stakeholder involvement and awareness [12,13]. While the reflections these papers provide are helpful, they do not report outcome data to support the success of implementation of the strategies that were employed, and descriptions of how stakeholder training for pharmacy accreditation self-studies were conducted are lacking. The lack of other studies describing how stakeholder interaction is encouraged during preparation indicates that there is a need for further research in this area.

Team Based Learning (TBL) is a constructivist pedagogical approach that has been successfully implemented in various Doctor of Pharmacy programs and in other educational settings [14,15,16,17]. Multiple studies have demonstrated that TBL pedagogy promotes student engagement [17,18,19,20]. Based on the premise that adult learners learn best by “doing”, it emphasizes immersing the learner in active engagement with the content through mandatory pre-reading, followed by in-class team activities (“application” exercises) that contextualize the content using “real-life” case-based scenarios that require the engagement of all team members. These team activities serve to ensure that participants understand the content and can apply it appropriately while identifying and correcting misconceptions. Importantly, this team approach leverages the knowledge and experiences of all team members and allows for diverse approaches to problems to be considered and discussed. This increases the sense of ownership by all participants and builds strong working relationships. While the ability of TBL to promote understanding and engagement has been well documented in an academic classroom setting [17,21,22], it is not frequently used in other settings and, to our knowledge, has not been used for accreditation self-study training purposes.

Based on research demonstrating that TBL pedagogy increases student engagement, we hypothesize that TBL constitutes an effective strategy for optimizing stakeholder engagement and an understanding of the accreditation process and requirements. We further propose that the use of TBL for accreditation self-study training will promote and enhance effective forming, norming, storming, and performing of teams of individuals, committees, and organizations that will participate in the accreditation process, all of which are essential and time-consuming stages of effective team development.

## 2. Materials and Methods

### 2.1. Research Participants

Data for this research study were obtained from participants in the ACPE accreditation self-study training workshop. Participants included administrators, faculty, staff, students, preceptors, and alumni. This study was approved by the California Northstate University (CNU) IRB committee (protocol #0513-01-27; approval date 6 January 2018). All faculty and staff members were required to attend the workshop. We invited all preceptors, students, and residents to attend via an e-mail in which we noted the importance of the self-study and the value of their input; however, we unable to make their attendance mandatory.

### 2.2. Overview of the Use of Team-Based Learning Pedagogy within the Accreditation Self-Study Training Workshop

**Figure 1:**
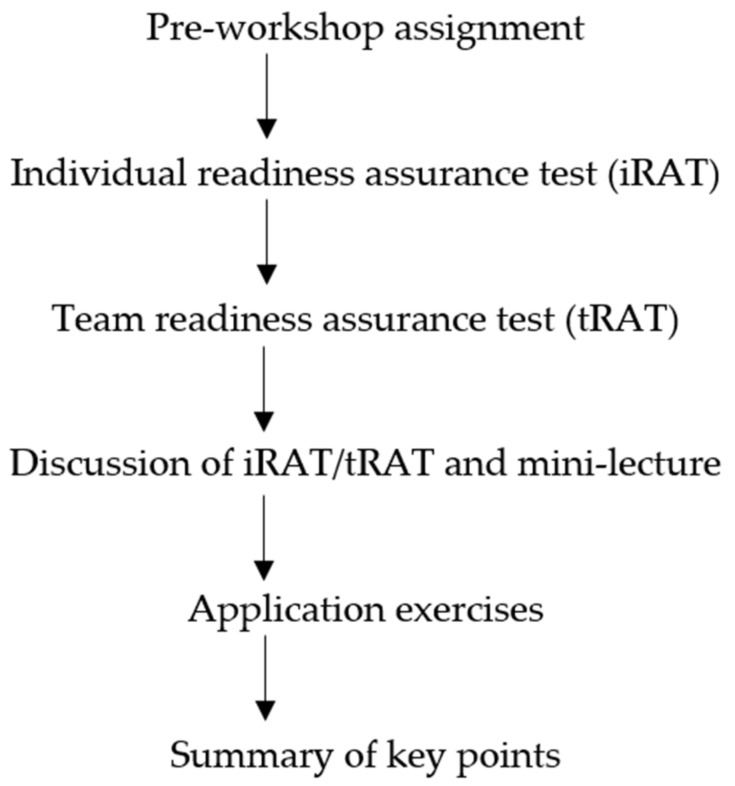
Overview of the Team-Based Learning (TBL) process used for the accreditation self-study workshop.

The accreditation self-study workshop was conducted onsite at CNU College of Pharmacy (CNUCOP) in 2018. Dr. Ruth Vinall, a Team Based Learning Collaborative (TBLC) trainer-consultant, led the workshop and it lasted for 3 h. All CNUCOP faculty and staff were required to attend the workshop. All students, preceptors, and residents were invited to attend via e-mail. While students, alumni, preceptors, and residents were not required to attend, we emphasized the importance of the self-study to them and emphasized that their participation would be highly valued as they are key stakeholders. Participants were notified that Team-Based Learning (TBL) pedagogy would be employed for the accreditation self-study training and were asked to complete an assignment prior to attending the workshop, per the TBL format (Figure 1). This assignment stated the objectives of the workshop and why the workshop was important and contained the following information: steering committee and subcommittee member composition, assignment of accreditation standards, an overview of the standards, evidence required for each standard, and the ACPE self-evaluation rubric and definitions. The pre-workshop assignment clearly stated what participants were expected to be able to do prior to the workshop, and participants were told that there would be a pop quiz (individual readiness assurance test, iRAT) at the start of the workshop based on these expectations. The assignment was e-mailed to participants one week before the workshop.

At the start of the 2 h workshop, participants were asked to sit at tables with their team members (from four to six members per team). Teams were assigned based on what ACPE standard subcommittee stakeholders were asked to serve on. For example, subcommittee 1 (assigned to work on ACPE standards 1, 5–9, and 16) members were assigned to the same team. There were a similar number of faculty, staff, preceptors, residents, and students on each team. Each participant was then asked to complete an iRAT (five multiple choice questions) without receiving help from team members/looking up answers. The iRAT is administered at the start of a TBL class to encourage participants to complete the pre-class assignment and thereby give them a foundational knowledge of the topics that will be covered in the mini-lecture and team application exercises. It was used in a similar fashion for our accreditation self-study workshop to encourage participants to review workshop materials prior to attending the workshop. Each team then worked together to answer the same questions using Immediate Feedback Assessment Technique (IF-AT) cards (Epstein Educational Enterprises, Cincinnati, OH). These are scratch cards, similar in style to lotto tickets, which reveal a star symbol if the correct answer box is scratched off. IF-AT cards have been shown to stimulate engagement in an educational setting, most likely because they are similar in nature to lotto tickets and provide a sense of excitement [23]. Teams were allowed 5 min to complete the iRAT and an additional 5 min to complete the team readiness assurance test (tRAT, the term given to the component in which teams use IF-AT cards to collectively answer questions). Participants were then asked if they had questions or concerns or wanted to further discuss contents of the pre-workshop assignment, and key points were emphasized and further explained as part of a mini-lecture. The iRAT component encourages participants to complete the pre-class assignment while the tRAT and post-tRAT group discussion components encourage discussion and the identification and correction of any misconceptions relating to the pre-workshop materials. Next, participants worked on application exercises with their teams; they were given one hour to answer scenario-based questions. In contrast to the iRATs/tRATs, which simply test remembering and understanding of content, TBL application exercises require higher-level thinking skills such as applying, analyzing, and evaluating, all of which are important for completion of the accreditation self-study process. Working on application exercises also helps teams get to know each other and build strong working relationships. After completion of the application exercises, teams were asked to present and explain their answers to all participants and inter-team discussion was encouraged. This inter-team discussion provided further opportunities for any misconceptions to be identified and addressed and for concerns to be raised and dealt with in a timely manner. The training session concluded with a summary of key points and considerations and next steps.

### 2.3. Survey Administration and Data Collection

All ACPE accreditation self-study training workshop participants were invited to participate in the study. A survey comprising 17 Likert scale questions was administered by SurveyMonkey at the end of the workshop (Table 1). This survey was based on a survey generated by Lein et al. that was used to assess the utility of TBL in a classroom setting [24]. Participants were also asked to state how many self-studies they had previously participated in. Responses were voluntary, anonymous, and submitted electronically.

### 2.4. Data Analyses

Data were analyzed with IBM SPSS Statistics (Version 26). The Likert-scale questionnaire was divided in two parts, the first from question 1 to 12 and the second from question 13 to 17. Internal consistency for each section was evaluated using Cronbach’s alpha (α > 0.70 was considered acceptable). Participants were divided between attendees that had already been in an accreditation self-study and the those who were first timers. Answers from these two groups were analyzed using a Mann–Whitney U test and an independent-samples median test (*p* < 0.05 was considered significant).

## 3. Results

A total of 52 people (32 faculty members out of a total of 38 faculty members, 9 staff out of a total of 11, 2 preceptors out of a total of 141, 7 students out of a total of approximately 400, and 2 residents out of a total of 4) attended the accreditation self-study workshop. Of these, 30 were male and 22 were female. Twenty-four of these people (46%) completed an online survey after completion of the workshop, 12 of whom (50%) had previously participated in at least one accreditation self-study.

### 3.1. Responses to Questions Relating to Participant Engagement and Usefulness of Team Activities

**Figure 2:**
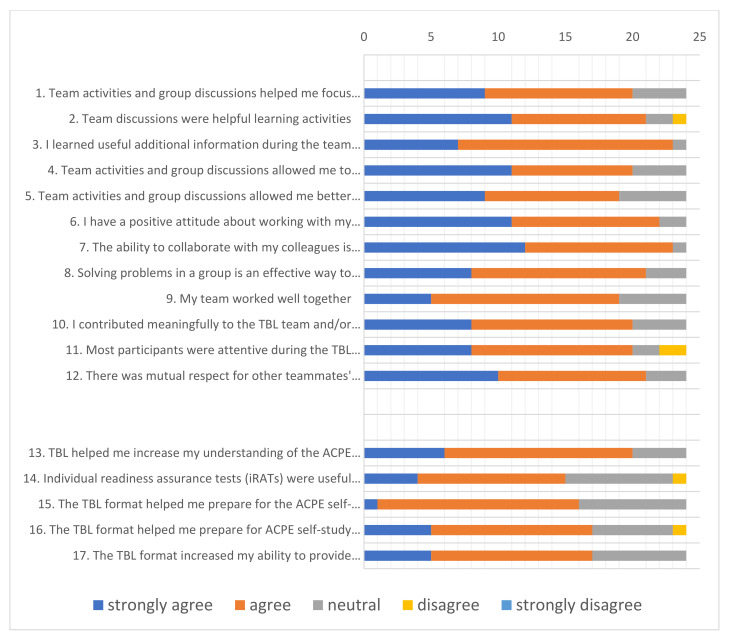
Survey data. A total of 24 workshop attendees participated in the survey study. These participants provided answers to all of the 17 questions.

Twelve of the survey questions related to participant perceptions of self and team member engagement in the team activities and the usefulness of the team activities in terms of preparing them for the accreditation self-study (Figure 2, first 12 questions). This component of the survey had high internal consistency with a Cronbach’s alpha of 0.94.

Our data indicate that the team activities effectively engaged the majority of the self-study workshop participants; 83% of participants strongly agreed or agreed that they made meaningful contributions to team discussions, and 92% strongly agreed or agreed that they had a positive attitude about working with colleagues in this setting. Effective engagement was further confirmed by the high number of participants who strongly agreed or agreed that most participants were attentive during activities and that their team worked well together (83% and 79%, respectively).

Our data also indicate that the majority of participants found the team activities helpful in preparing them for the accreditation self-study: 96% of participants strongly agreed or agreed that they learned useful information and 87%, 84%, 79%, and 83% strongly agreed or agreed that team discussions were helpful learning activities, allowed them to better understand the process, helped them to focus on important issues, and helped them to correct misconceptions, respectively. The latter is very important because misconceptions are often difficult to identify and correct and can lead to significant confusion and frustration during the self-study process. Our data indicate that participants also supported the rationale for using a team approach to self-study training; 88% strongly agreed or agreed that solving problems as a group was an effective way to prepare for an accreditation self-study, and 96% strongly agreed or agreed that the ability to collaborate with colleagues is necessary for the self-study process. Lastly, subgroup analyses based on whether participants had or had not previously worked on an accreditation self-study (*n* = 12 for each group) revealed no differences between the two groups, indicating that people with and without self-study experience found the team-based approach to the workshop beneficial.

The combined data demonstrate that using team activities for an accreditation self-study workshop can result in high levels of participation and is a helpful way to prepare people for an accreditation self-study. They also demonstrate that team activities are effective in a setting where not everyone has prior experience, which is a common occurrence for accreditation self-studies.

### 3.2. Responses to Questions Relating to the TBL Format

Five of the survey questions related to how helpful participants found the TBL approach, including the individual readiness assurance test (iRAT) (Figure 2, last five questions). This part of the survey also had high internal consistency with a Cronbach’s alpha of 0.86.

Our data indicate that the TBL format was well received by the majority of participants: 66%, 84%, 71%, and 71% strongly agreed or agreed that the TBL format helped them prepare for the self-study, helped increase understanding of the self-study process, increased their ability to provide meaningful contributions to discussions, and helped them prepare for the self-study voting process, respectively. Understanding the voting process is a very important aspect of a self-study, as there are specific and somewhat unintuitive meanings to the voting options for each of the self-study standards (‘compliant’, ‘compliant with monitoring’, ‘partially compliant’, or ‘non-compliant’). Our data show that 63% of participants strongly agreed or agreed that the iRAT was a helpful learning activity. While this percentage is high, this was the lowest scoring item on the survey suggesting these individual quizzes were perceived to be less helpful than the TBL team activities. Lastly, subgroup analyses revealed no differences between participants who had previously participated in an accreditation self-study versus those who had not, again indicating that the TBL approach was beneficial regardless of self-study experience.

Our combined data indicate that implementation of the TBL format within an accreditation self-study workshop can be an effective way to help prepare participants for a self-study and demonstrate that TBL pedagogy has utility in a non-educational setting.

### 3.3. Study Limitations

Only 46% of workshop participants completed the survey, meaning that the data may not be truly representative of the group. The sample size (*n* = 24) was also small, although it should be noted that this was an exploratory study and only descriptive statistics, not inferential statistics, were reported. Another limitation is that this study only included data from a single student cohort at a single college of pharmacy, and as such it is not certain that similar results would be observed at other colleges of pharmacy. Lastly, while Cronbach alpha values indicate our survey data had high internal consistency, the survey used has not been formally validated. Larger multi-site studies will be needed to validate our findings.

## 4. Discussion

Our data demonstrate that implementation of Team Based Learning (TBL) pedagogy in a ‘kick-off’ workshop to prepare stakeholders for an accreditation self-study can result in high levels of engagement and was perceived as helpful by workshop attendees. To our knowledge, TBL pedagogy has not previously been used in this setting, and the benefits of implementing team activities as part of stakeholder training have not previously been reported. While this study was exploratory in nature, our data indicate that TBL pedagogy may be useful in settings outside of the classroom, indicating that larger, multi-site studies are warranted.

It is widely accepted that team activities promote engagement and the improved retention and understanding of important concepts and processes in an educational setting. As such, they are a key component of many teaching pedagogies, including TBL [21,22,25]. Team activities are also frequently implemented in workplace settings, although often the primary goal of these is to enhance camaraderie and trust between team members, and the activities may not directly relate to job function (i.e., they simply serve as ‘team-building’ activities). In our study, TBL was used outside of a traditional academic classroom setting to provide specific training to stakeholders from various backgrounds (faculty, staff, students, preceptors, alumni). The benefits of providing task-oriented training in a team setting at the beginning of projects has been clearly demonstrated by McEwan et al. [26]. Their meta-analysis (51 independent studies, *n* = 8439) demonstrated that providing training in a team format at the start of a project increased performance not only in academic settings but also in the fields of healthcare and aviation and the military. Importantly, they found training that included team activities resulted in much better performance compared to training which included only didactic lectures. While we did not assess performance in our study, the data from this meta-analysis, combined with our finding that participants were engaged and found the team activities helpful, strongly indicate that providing training through team activities is likely to be beneficial for the accreditation self-study process. Again, to our knowledge, the benefits of including team activities as part of accreditation self-study preparation has not previously been reported.

The accreditation self-study process involves multiple stakeholders and is extremely time-consuming and somewhat complex, so providing adequate and effective training is key to maximize efficiency and minimize frustrations. An overview of how accreditation self-studies are conducted is a required component of ACPE self-study reports, many of which can be found online. Per our review of more than 20 publicly available self-study reports (found by entering the following search terms into Google; ‘ACPE self-study’, ‘ACPE’, ACPE accreditation’, ‘ACPE accreditation report’) it appears that many colleges provide an informational session and/or some type of training to stakeholders at the start of a self-study; however, none of the reports mentioned the use of team activities or TBL. We were also unable to find any mention of the use of team activities or TBL for accreditation self-study preparation in our review of the literature (PubMed search). Many of the publicly available self-study reports stated that informational ‘kick-off’ meetings were held for stakeholders in which self-study steering committee members provided an overview of the process and expectations. For example, the University of Houston College of Pharmacy self-study document states that “the Dean and self-study co-chairs reviewed the purpose and design of the self-study with the attendees” [27]. Some colleges reported assigning stakeholders to committees and letting individual committee chairs provide standard-specific information to their members. For example, the Chicago State University College of Pharmacy self-study document states that “each member of the Self-Study Committee was asked to chair/co-chair a subcommittee to address the development of each standard” [28]. Our study data indicate that including team activities and using a TBL format promotes engagement and is considered helpful by participants for accreditation self-study preparation. Our data align with findings from TBL research studies conducted in an educational setting which also demonstrated that implementation of TBL resulted in increased engagement [14,15,16,17]. Other benefits of using TBL include that it promotes knowledge retention, collaboration, and a sense of shared responsibility [21,22]. While these factors were not assessed in our study, they are important because most self-studies take at least one year to complete and involve continued collaboration between multiple stakeholders with very different backgrounds who may or may not have prior experience with self-studies. Our subgroup analyses showed no differences in perceptions of engagement and usefulness of TBL from stakeholders who had previously been involved in a self-study versus those who had not. This is encouraging, as it indicates that use of TBL for self-study preparation compares favorably with past experiences, and that it is also useful to people who are new to the process.

While TBL has not previously been used for accreditation self-study preparation, it has been used successfully for other training that occurs outside of the traditional academic classroom setting. For example, the Boy Scouts of America organization have used TBL to conduct their executive training workshops, and in doing so were able to reduce training from five to two days [29]. A similar educational pedagogy, problem-based learning (PBL), which also employs team activities to promote engagement and learning, has also been used to conduct training outside of an academic classroom setting. For example, PBL has been used to train police academy students, and was found to improve critical thinking skills compared to traditional lecture-based training [30]. It is noteworthy that while both TBL and PBL include team activities, there are several key differences between them [31]. For example, in PBL each team is assigned a moderator, while in TBL all teams are typically supervised by one instructor, meaning that less feedback may be received. There is also no iRAT/tRAT equivalent in PBL. The combined data indicate that TBL as well as PBL has utility outside of a traditional academic classroom setting and suggest the broadened usage of educational pedagogies that promote active learning is warranted.

The component of the training that participants in our study found the least beneficial was the individual readiness assurance test (iRAT). The iRAT is designed to encourage participants to complete a pre-class assignment and thereby come to class (in this case the workshop) with a foundational knowledge of the topic(s) that will be covered. Our finding that some participants did not find iRATs beneficial aligns with educational studies conducted in a classroom setting. Some students also stated that they did not find iRATs helpful [32], which may in part relate the fact that completing pre-class work and being held accountable for completion of pre-class work are not generally required components of other teaching pedagogies and can be time consuming and challenging. It is likely that, as with traditional students, some participants found iRATs frustrating and/or an inconvenience, especially if they had not completed the pre-class assignment. Further studies would be needed to determine the exact reasons. While the pre-class assignments and iRATs may not be deemed helpful by all TBL participants, a major benefit of iRATs (followed by tRATs in which teams discussed then answered the same questions) is that they allow for key facts and fundamental principles to be emphasized and allow for more time to be spent on team activities, as extensive review of informational material during class time is not required. This is important because it is these activities which best promote engagement with and understanding of higher-level concepts and provide practice with using the information learned as well as recognition and correction of misconceptions. It is likely that explaining the purpose and utility of iRATs to participants prior to implementation of TBL within a self-study training setting may help mitigate the concerns of some participants.

In conclusion, our data support the usage of TBL for accreditation self-study training. TBL pedagogy has been successfully used for many years in an educational setting to promote student engagement and help students from diverse backgrounds work together to solve complex problems and complete tasks. Our study data show that stakeholders in an ACPE accreditation self-study, many of whom were also from diverse backgrounds, found TBL pedagogy engaging and helpful in preparing for the self-study process, and our study thereby supports further investigations into the value of TBL pedagogy in this setting.

## Figures and Tables

**Table 1:** Survey questions. The survey comprised 17 questions. The first 12 questions relate to participant engagement and usefulness of the workshop activities, the last five questions relate specifically to the usefulness of the TBL format.

Question Number	Question Text (Answer Options for All Questions ‘Strongly Agree’, ‘Agree’, ‘Neutral’, ‘Disagree’, or ‘Strongly Disagree’)
1	Team activities and group discussions helped me focus on important information
2	Team discussions were helpful learning activities
3	I learned useful additional information during the team activities and group discussions
4	Team activities and group discussions allowed me to correct misunderstandings
5	Team activities and group discussions allowed me to better understand the ACPE self-study process and materials
6	I have a positive attitude about working with my colleagues on the ACPE self-study process
7	The ability to collaborate with my colleagues is necessary for the ACPE self-study to be successful
8	Solving problems in a group is an effective way to prepare for the ACPE self-study process and voting
9	My team worked well together
10	I contributed meaningfully to the TBL team and/or group discussions
11	Most participants were attentive during the TBL session(s)
12	There was mutual respect for other teammates’ viewpoints during the TBL session(s)
13	TBL helped me increase my understanding of the ACPE self-study process and materials
14	Individual readiness assurance tests (iRATs) were useful learning activities
15	The TBL format helped me prepare for the ACPE self-study process
16	The TBL format helped me prepare for ACPE self-study voting
17	The TBL format increased my ability to provide meaning contributions to the ACPE self-study process
